# Foam 3D Printing of Thermoplastics: A Symbiosis of Additive Manufacturing and Foaming Technology

**DOI:** 10.1002/advs.202105701

**Published:** 2022-02-20

**Authors:** Mohammadreza Nofar, Julia Utz, Nico Geis, Volker Altstädt, Holger Ruckdäschel

**Affiliations:** ^1^ Sustainable and Green Plastics Laboratory Metallurgical and Materials Engineering Department Faculty of Chemical and Metallurgical Engineering Istanbul Technical University Istanbul 34469 Turkey; ^2^ Polymer Science and Technology Program Istanbul Technical University Maslak Istanbul 34469 Turkey; ^3^ Department of Polymer Engineering University of Bayreuth Bayreuth 95447 Germany; ^4^ Bavarian Polymer Institute and Bayreuth Institute of Macromolecular Research University of Bayreuth Bayreuth 95447 Germany

**Keywords:** 3D printing, additive manufacturing, foaming, microcellular foams, reviews, thermoplastics

## Abstract

Due to their light‐weight and cost‐effectiveness, cellular thermoplastic foams are considered as important engineering materials. On the other hand, additive manufacturing or 3D printing is one of the emerging and fastest growing manufacturing technologies due to its advantages such as design freedom and tool‐less production. Nowadays, 3D printing of polymer compounds is mostly limited to manufacturing of solid parts. In this context, a merged foaming and printing technology can introduce a great alternative for the currently used foam manufacturing technologies such as foam injection molding. This perspective review article tackles the attempts taken toward initiating this novel technology to simultaneously foam and print thermoplastics. After explaining the basics of polymer foaming and additive manufacturing, this article classifies different attempts that have been made toward generating foamed printed structures while highlighting their challenges. These attempts are clustered into 1) architected porous structures, 2) syntactic foaming, 3) post‐foaming of printed parts, and eventually 4) printing of blowing agents saturated filaments. Among these, the latest approach is the most practical route although it has not been thoroughly studied yet. A filament free approach that can be introduced as a potential strategy to unlock the difficulties to produce printed foam structures is also proposed.

## Graphical abstract

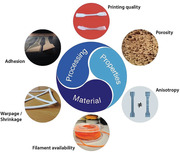

## 1 Introduction

**Figure 1:**
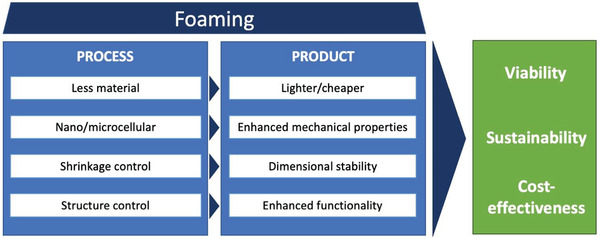
Advantages of foaming from the processing, property, and product perspectives.

Foaming of thermoplastics results in more sustainable, lighter, and less expensive components for a variety of commodity and engineering applications. This is while the foamed structures encounter less shrinkage and better dimensional stability due to the lower material input.^[^ ^1^ ^]^ On the other hand, foamed products have better weight‐related properties, such as specific impact strength and toughness, compared to their solid counterparts. Foaming could also offer significant thermal and acoustic insulation properties. Noticeable improvements in the specific properties can be achieved by controlling the cellular structure.^[^ ^2^ ^]^ Functional properties could also be induced in foamed components, for instance by using electrically conductive nanoparticles such as carbon nanotubes or graphene, as well as barrier‐forming nanoclay or cellulose nanocrystals.^[^ ^3^ ^]^ **Figure** **1** summarizes the product‐ and process‐related advantages that can be associated with cellular foam structures.^[^ ^4^ ^]^ These unique features are the motivation to further develop and transfer advanced foaming technologies to a wider range of applications.

**Figure 2:**
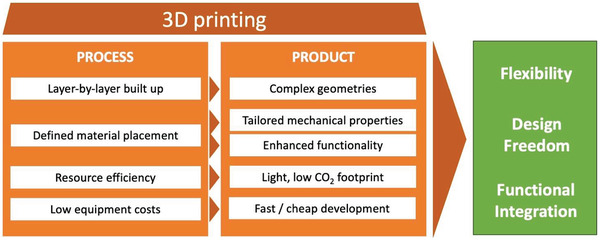
Advantages of 3D printing from the processing, property, and product perspectives.

Additive manufacturing or 3D printing is also a technology to produce defined structures and geometries. This method of layer‐by‐layer built‐up enabling the fabrication of parts with complex geometries, is a rapidly growing technology for many fields of applications due to its various advantages some of which are presented in **Figure** **2**.^[^ ^5^ ^]^ A very important benefit of additive manufacturing is that it does not require expensive tooling like in injection molding. This allows a new freedom regarding design and complexity of components and leads to a more time‐ and cost‐efficient development of new products.^[^ ^6^ ^]^ The ability to use material exactly where it is needed and to combine different materials in one part makes it possible to tailor properties such as mechanical, thermal, or electrical conductivity. This results in products with high functionality, individual design and, similar to foaming, reduced weight.^[^ ^7^ ^]^ Moreover, as 3D printers can manufacture a product directly from a computer‐aided design (CAD) model, the time between completion or modification of the model and the real part is extremely short. This is highly beneficial for prototyping and production of replacement parts.^[^ ^7a^ ^]^

So far, the production of thermoplastic polymer foams was based on manufacturing methods such as extrusion foaming,^[^ ^8^ ^]^ foam injection molding,^[^ ^9^ ^]^ bead foaming,^[^ ^10^ ^]^ rotational foam molding,^[^ ^11^ ^]^ and compression foam molding.^[^ ^12^ ^]^ Among these, extrusion foaming, foam injection molding, and bead foaming have been often preferred due to their higher productivity and are nowadays widely used to produce various foamed structures.^[^ ^13^ ^]^ In addition to these common foaming technologies, additive manufacturing could also be integrated as an innovative foam manufacturing technology. Simultaneous foaming and 3D printing (i.e., foam 3D printing), which is not yet well established, is expected to be a rapidly growing research area and industrial trend as a promising alternative to foam injection molding technology. In contrast to foam injection molding which requires a serious capital cost, the advantages of 3D printing technology such as design freedom, tool‐less production, and instant manufacturing could make it a more affordable foam manufacturing approach for small and medium‐sized companies, and even more cost‐effective for large‐sized companies. This perspective review article classifies attempts of integrating foaming technology into 3D printing and explains the challenge involved. Efforts to combine these technologies have been clustered into 1) architected porous structures, 2) syntactic foaming, 3) post‐foaming of pre‐manufactured printed parts, and finally 4) printing filaments containing blowing agents. Among the aforementioned attempts, the latest approach seems to be a successful practical route to simultaneously manufacture printed foam structures although it has not been thoroughly studied yet. This review also proposes a filament free approach as a potentially new and more practical foam 3D printing route.

## 2 Foaming and Additive Manufacturing as Separate Technologies

### 2.1 Polymer Foams and Foaming Technology

**Figure 3:**
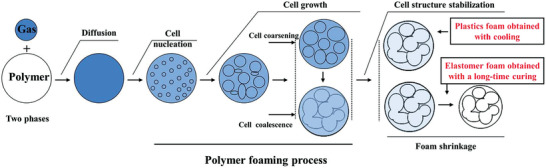
Schematic of formation of polymer/gas solution, cell nucleation, cell growth, and cell structure stabilization during polymer foaming. Reproduced with permission.^[^ ^17^ ^]^ Copyright 2021, Taylor & Friends.

Polymer foams generally consist of a solid polymer matrix and a gaseous phase that contributes to the formation of cells within the polymer structure.^[^ ^1b^ ^]^ In thermoplastic polymer foams, the cellular structure is formed by impregnation with a blowing agent which is either a physical blowing agent (PBA) or a chemical blowing agent (CBA). The dissolved blowing agent generates gas during foaming process and subsequently foaming occurs due to a thermodynamic instability caused by the supersaturation of the blowing agent (i.e., either a pressure drop or a temperature increase).^[^ ^14^ ^]^ Such thermodynamic instability during foaming causes cell nucleation and continues in cell growth. During cell growth depending on the rheological properties and crystallization behavior of the polymer/gas mixture, coarsening or coalescence of the cells may occur, which is usually unfavorable.^[^ ^15^ ^]^ The foam structure is then produced by expulsion of the dissolved blowing agent from the polymer/gas mixture and foam products are produced with cell stabilization as the pressure and temperature reaches the ambient condition.^[^ ^1^ ^]^ In thermoplastic elastomers the foam is unlikely to shrink during cell stabilization. Such shrinkage could be controlled by the degree of crystallinity in thermoplastic elastomers^[^ ^16^ ^]^ or the curing mechanism and degree of vulcanization in thermoset elastomers.^[^ ^17^ ^]^ **Figure** **3** schematically illustrates the steps involved in polymer foaming when a gas is used as a blowing agent.

#### 2.1.1 Classification of Polymer Foams

**Figure 4:**
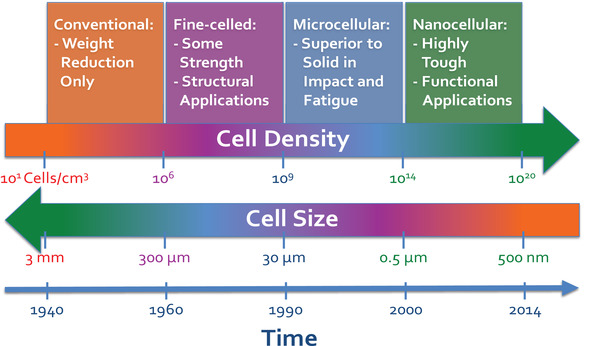
Classification of polymer foams based on their cell density and cell size over time. Reproduced with permission.^[^ ^1a^ ^]^ Copyright 2017, Elsevier.

Thermoplastic foams can be categorized by their cell size, foam density, and cell structure. According to the cell size and cell population density, thermoplastic polymer foams are classified into conventional, fine‐celled, microcellular, and nanocellular foams.^[^ ^1^, ^18^ ^]^ **Figure** **4** shows the classification of polymer foams based on their cell size and cell population density over time. Microcellular polymer foams, that is, foams with cell size less than 30 µm and cell density in the range of 10^9^–10^12^ cells/cm^3^, have been widely commercialized in industry to manufacture products for various commodity and engineering applications.^[^ ^1^ ^]^ Due to their unique cellular structure, microcellular foams can exhibit high impact strength and toughness compared to their solid counterparts while they can also reveal a good light reflecting‐ability.^[^ ^19^ ^]^ In the last decade, nanocellular polymer foams (structures with a cell size below 1 µm and cell density of more than 10^12^ cells/cm^3^) have attracted a lot of attention due to their outstanding features such as superthermal insulation.^[^ ^20^ ^]^

Depending on their density and void fraction, thermoplastic polymer foams can also be classified as: high density foams (i.e., less than 4 times expansion), medium density foams (i.e., between 4 and 10 times expansion), low density foams (i.e., between 10 and 40 times expansion), and ultralow density foams (i.e., more than 40 times expansion). High density foams are mainly used for construction materials, furniture, transportation, and automotive products. These foams are usually produced by foam injection molding when complex 3D geometries are required. Low density foams are mainly used for impact absorption, sound insulation, and packaging materials and are mostly produced by either extrusion foaming when continuous profile structures are needed or bead foaming when complex 3D geometries are designed.^[^ ^1^, ^8^ ^]^

Thermoplastic polymer foams can also be categorized based on their cell structure whether they are open‐cell or closed‐cell. Open‐cell foams are interconnected structures, while closed‐cell foams contain no openings between their cell walls. Since each of these structures provides different properties, they can be incorporated in various applications. For instance, open‐cell foams are utilized in sound insulation or filtering applications whereas closed‐cell foams are used for structural applications or heat insulation.^[^ ^1^, ^8^ ^]^

#### 2.1.2 Foam Manufacturing Technologies

Extrusion foaming, foam injection molding, and bead foaming are three commonly employed technologies for processing thermoplastic foam. In extrusion foaming, one of the main advantages is the production of low density polymer foams with continuous profile and simple 2D geometries.^[^ ^21^ ^]^ In contrast to foam extrusion, foam injection molding is usually used to manufacture high‐density foam structures with complex 3D geometries.^[^ ^21^ ^]^ The final foam injection molded part results in a product with lower material cost, high dimensional stability, lower energy consumption, and a shorter cycle time. Bead foaming is another common alternative to produce low‐density foam products with 3D geometries. This method involves the manufacturing of low‐density bead foams followed by steam chest molding into the desired final shape.^[^ ^10^, ^22^ ^]^

#### 2.1.3 Foam Blowing Agents

Most thermoplastic foams can be blown with either a CBA or a PBA. CBAs are solid substances that can decompose exothermically and endothermically during the foam processing at elevated temperatures. Hence, they generate gases like CO_2_ and/or N_2_ during the melt processing and a polymer/gas mixture can be formed during decomposition of these agents. PBAs are, however, materials that are injected into the polymer melt during the process in either a liquid, gaseous, or supercritical phase. N_2_ or CO_2_ are commonly used PBAs which dissolve in the polymer melt as gas or supercritical phase. By creating thermodynamic instability, phase separation occurs and causes the foaming. Among these inert gases, N_2_ has much lower solubility and higher diffusivity than CO_2_. This property of N_2_ makes it more advantageous in the production of foams with higher cell density with low expansion ratios (i.e., high‐density foams) and is mainly used in foam injection molding technology. This is because N_2_ has a higher cell nucleation capacity than CO_2_ due to its higher diffusivity. In contrast, in foam technologies such as foam extrusion, where larger expansions (i.e., low‐density foams) are required, CO_2_ can be used due to its higher solubility in molten polymers. Hydrocarbons such as pentane also have a low boiling temperature and are present in the polymer melt as a liquid under elevated pressures.^[^ ^21^, ^23^ ^]^ During depressurization, these blowing agents immediately change from liquid to gaseous and cause cell nucleation and growth. Although the use of hydrocarbons leads to the formation of foams with high expansions as a result of their high solubility and low diffusivity in polymers, they are not preferred nowadays due to their toxicity and flammability.^[^ ^18^ ^]^

### 2.2 Additive Manufacturing

#### 2.2.1 3D Printing Methods

The terms “3D printing” or “additive manufacturing” describe shaping processes, where components are built up layer‐by‐layer based on a CAD model. There is a multitude of technologies for manufacturing components based on starting materials in the solid, liquid, or gaseous phase. Important methods for polymer‐based materials are divided into four groups, with each group containing different technologies.^[^ ^24^ ^]^ The first group is powder bed fusion technologies, in which the polymer is applied to the printing bed as a powder layer and fused using various methods. In multi jet fusion (MJF), the powder is melted by infrared light. Locally applied binders cause a change in thermal conductivity and thus a selective fusion of the powder.^[^ ^25^ ^]^ The second method, selective laser sintering (SLS), uses a laser to sinter/melt the polymer powder locally.^[^ ^26^ ^]^ Other technologies are based on material extrusion. The most popular technology is fused filament fabrication (FFF), also known as fused deposition modeling (FDM).^[^ ^27^ ^]^ The polymer is fed to the printer as a filament, melted in a printing head and deposited on a printing platform. There is also the option of extruding directly from granulate using direct material extrusion. These technologies are known as fused granulate fabrication (FGF), fused particular fabrication, or pellet printing.^[^ ^28^ ^]^ A special method is ARBURG Plastic Freeforming. In this case there is no continuous extrusion of the melt, but a piezo actuator controls the droplet‐wise deposition of the material.^[^ ^29^ ^]^ Another concept is material jetting,^[^ ^30^ ^]^ where viscous droplets of photopolymers are applied and cross‐linked by UV light. The fourth group of 3D printing technologies also deals with photopolymerization. The photocurable materials are stored in a resin container and cured layer‐by‐layer by selective application of light energy. The light source can be either a laser in stereolithography or a projector in digital light processing.^[^ ^31^ ^]^ For thermoplastics, today MJF, SLS, and FFF are mainly used. Due to the superior relevance of FFF for producing foamed 3D printed materials, this technology will be described in more detail.

#### 2.2.2 Fused Filament Fabrication

**Figure 5:**
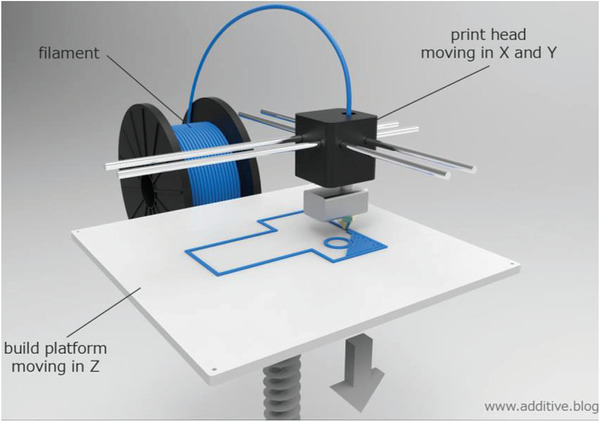
Principle of an FFF process. Reproduced with permission.^[^ ^38^ ^]^ Copyright 2021, Additive Blog.

FFF is one of the most widely used additive manufacturing technologies, originally invented by the company Stratasys in the 1980s^[^ ^32^ ^]^ and further developed by the RepRap project community to accelerate the development of this technology.^[^ ^33^ ^]^ As FFF printers are desktop devices and affordable, an increasing number of companies and consumers started to use FFF printing. To date, many different FFF printers and new materials have entered the market and expanded the application areas.^[^ ^27^, ^34^ ^]^ The principle of the FFF process, which is one of the melt‐based additive manufacturing concepts, is shown in **Figure** **5**. A continuous thermoplastic filament is melted in a printing head and deposited in thin lines on the build platform. A layer is built by moving the printing head on rods in *x*‐ and *y*‐directions. Via movement of the build platform in *z*‐direction a printed part is built up layer‐by‐layer.^[^ ^7^, ^35^ ^]^ The printer's movements are controlled by the G‐code generated by a slicing software, which calculates the layer structure based on a CAD model and different process parameters such as layer height and infill pattern.^[^ ^36^ ^]^ In addition, material parameters are handled by the slicing software. The challenge of slicing is to find suitable parameters for each combination of printer, part geometry, and material to achieve a good printing quality. The most important parameters that can be set are filament diameter, nozzle temperature, temperature of build platform, printing speed, cooling speed, layer height, line width, infill degree, infill pattern, and orientation.^[^ ^37^ ^]^

#### 2.2.3 Application of FFF 3D Printed Thermoplastic Structures

FFF is an affordable and fast technology to transfer an idea into a real part. Therefore, it is frequently used in education to illustrate geometries, help manufacturing low‐cost scientific equipment, or put student projects into practice. It is also a very helpful and fast method to design and produce individualized tools, jigs, and fixtures for easier handling, higher productivity, or more safety in industry.^[^ ^39^, ^40^ ^]^ FFF can be practically employed in a wide range of applications from toys to home gadgets and high‐tech products.^[^ ^41^ ^]^ Another important application is prototyping. Since neither expensive tooling nor pre‐ or post‐processing is required, new parts can be printed within hours. Design and function can be verified in a superior assembly and modifications can be made immediately. Hence, 3D printing is used across all industries for time‐ and cost‐saving in‐house prototyping. Another application area is the medical industry. The FFF process is used for guides and tools in surgery and for the fabrication of anatomical replicas for learning purposes.^[^ ^24a^ ^]^ Moreover, individualized orthoses, prostheses, or implants can be printed based on 3D scans or tomographic models.^[^ ^42^ ^]^ Customization also plays a role in sectors such as art and jewelry, consumer goods, or sports, where unique pieces with complex designs can be printed.^[^ ^43^ ^]^ Another field of application can be found in construction and architecture.^[^ ^44^ ^]^ On the one hand, additive manufacturing helps to build up and optimize models, on the other hand, it offers the possibility to create buildings and elements with new designs, high complexity, greater functional integration, and less waste. The high design freedom and possibility of complex geometries are also used for the design of new lightweight components, which are extensively used in aerospace industry.^[^ ^45^ ^]^

#### 2.2.4 Challenges of FFF Printing

**Figure 6:**
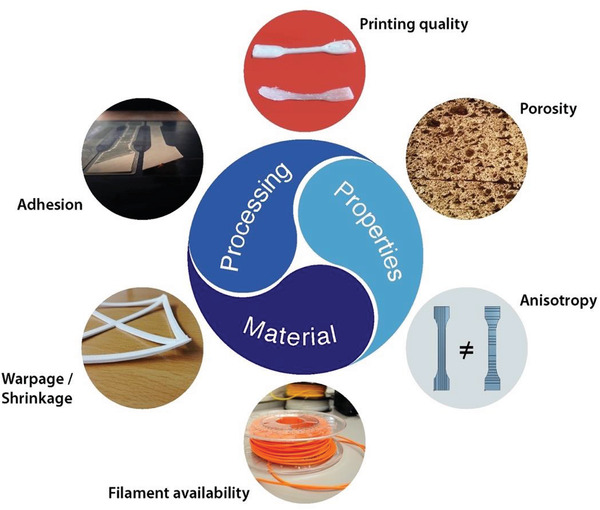
Challenges of FFF process.

Despite all its advantages, the FFF process still has some challenges to overcome in order to expand the range of applications. Essentially, all current problems are based on a tension that arises between material, process, and the properties of the finished part, as shown in **Figure** **6**.^[^ ^46^ ^]^ The requirements for a good printing material differ from the properties of the materials commonly used in other technologies. Currently, there are some materials that show a very good printing behavior but have poor product performance like mechanical properties or thermal resistance. In contrast, well‐known materials with good properties are not suitable for printing, for instance, because of high warpage or shrinkage. Therefore, the market is still dominated by a limited number of thermoplastics such as polylactide (PLA), acrylonitrile butadiene styrene (ABS), or polyethylene terephthalate glycol (PETG). In contrast, polymers such as polypropylene or polyamide (PA), which are frequently used in other manufacturing technologies, are quite rare. In the future, extensive research needs to be done on technical and high performance polymers.^[^ ^41^ ^]^ In addition, although the printing and slicing processes give the user a lot of freedom, finding suitable process parameters can take quite some time.^[^ ^47^, ^48^ ^]^ There are many aspects that need to be considered and also many potential printing errors and problems. The following items can be seen as short summary of some key issues. Further information is beyond the scope of this article and can be found in the literature.^[^ ^49^ ^]^ A very important challenge in the FFF process is adhesion—on the print bed and between layers. A good adhesion on the build plate is essential to prevent warpage and detachment from the bed before the printing job is completed.^[^ ^50^ ^]^ The adhesion between layers, which is important to achieve good mechanical properties, depends mainly on the material type, nozzle temperature, cooling conditions, and printing direction.^[^ ^51^ ^]^ Another concern in FFF processes is that printed parts contain a certain porosity content which can have negative impact on the final mechanical performance. There is less material than originally planned, and furthermore the pores may act as defects where stress concentrations occur. This phenomenon may lead to rapid failure, especially in presence of tensile loads.^[^ ^52^ ^]^

## 3 Routes to Generate Cellular Structures by Additive Manufacturing

Cellular polymers are applied in manifold applications today. Their low weight combined with specific physical properties such as low thermal conductivity or sound absorption makes them unique engineering materials. Besides the polymer matrix, the density and cellular structure strongly influence the performance. Due to its ability to design various geometries, additive manufacturing promises to tailor cellular polymers. First, it is important to mention that there are two different types of porosity in the context of additive manufacturing. As first type, pores are generated as intrinsic phenomena by processing itself. Even if the infill content is set to 100%, places exist where no material is deposited during the layer‐by‐layer built‐up. Regarding the FFF process, more or less a stacking of cylinders with pores in between is observed. This type of porosity is generally undesirable and can be considered a defect. But there is also the possibility of intentionally introducing porosity to form defined cellular structures. Different geometries can serve functions such as insulation or lightweight construction. In this chapter only the defined structures will be discussed. To date, there are various routes reported to generate cellular structures by 3D printing: 1) architected porous structures, 2) syntactic foams, 3) post‐foaming of pre‐formed solid structures, and 4) in situ foaming of filaments containing blowing agent. These routes are explained in detail below.

### 3.1 Architected Porous Structures

**Figure 7:**
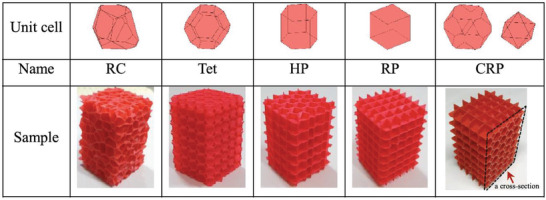
Overview of various unit cell designs by Wang et al.^[^ ^58^ ^]^ RC = Random Cel; Tet = Tetrakaidechedron; HP = Hexagonal Prism; RP = Rectangular Prism; CRP = Clipped Rectangular Prism. Reproduced with permission.^[^ ^58^ ^]^ Copyright 2017, Elsevier.

Considering architected porous structures in the context of additive manufacturing, porosity is recognized as a completely different concept. Components made from these structures are most likely built by adding unit cells. In principle, all additive manufacturing processes can be used to generate such structures. The “step‐by‐step” built‐up procedure by putting unit cells together, shows the main difference to foaming in its classical sense. Due to the high design freedom of additive manufacturing, there are almost no geometrical restrictions for the design of unit cells. As long as one uses a single extruder, the issues of the FFF process are overhangs or bridges. However, this undesired behavior can be solved by using a second extruder and a support material that is later dissolved. Therefore, various random and ordered geometries are feasible as shown by some structures in **Figure** **7**.^[^ ^53^ ^]^ By varying the smallest unit within a component, the overall properties of the final part can be controlled by the resulting scaffold topologies. In this context, the mechanical performance of such architected porous structures could be tailored by the designed geometry and unit cell assembly and its architecture.^[^ ^54^ ^]^ Hereby, it is possible to combine the high design freedom of 3D printing with the benefits of porous materials, especially in terms of their lightweight potential combined with their mechanical performance.^[^ ^55^ ^]^ Well‐known additive manufacturing processes allow us to access new fields of application. In medical technology, for example, prostheses, (bone) tissues, and (diabetes) insoles can be individually optimized and customized by architected porous structures.^[^ ^56^ ^]^ In literature, various shapes, sizes, and geometries of porous structures for numerous applications have been investigated.^[^ ^57^ ^]^ Overall, it can be said that the primary goal of 3D printed architected porous structures is an advanced functional integration, which can be perfectly achieved by controlling the key properties of unit cells. For instance, in tissue engineering, the porosity‐level must match the application in order to enable a suitable nutrient transport, which eventually allows an optimal tissue growth. Therefore, modeling is a powerful tool to create scaffold topologies that perfectly fit their application.

All results obtained in literature are highly dependent on the experimental setup and thus possess limited comparability. Therefore, it is necessary to use suitable simulation tools to make reliable predictions about the material performance. Moreover, the minimum dimensions of cells, cell walls, and cell struts are limited by the additive manufacturing process and are typically significantly larger compared to classical foaming technologies. In general, a key difference between this method and classical foaming is that the printed filaments are not porous structures but rather are built up on each other to produce final porous structures.

### 3.2 Syntactic Foaming

Syntactic foams are usually made of polymers containing hollow microspheres, for example, glass or polymer microspheres. Their density is comparatively large. Transferring the technology to additive manufacturing is straightforward. Microspheres could be easily integrated into the starting materials of additive manufacturing, for instance, thermoplastic filaments, and subsequently be 3D printed. There are some commercial materials addressing this topic. The company Lehmann&Voss&Co. provides a PA granular material for 3D printing that contains hollow glass spheres.^[^ ^59^ ^]^ In addition, Lay Filaments offers the Porolay filament series, whose filaments contain both an elastomeric component and PVA. Since PVA can be dissolved in water after printing, porous structures are formed.^[^ ^60^ ^]^

In literature, the group of Doddamani produced cellular 3D printed structures. They introduced the production of syntactic foams in which closed cell structures could be produced using hollow micro‐balloons compounded with thermoplastic matrices.^[^ ^61^ ^]^ They claimed that the entrapped hollow spheres were retained in the produced filaments as well as in the 3D printed samples. They introduced printed structures with void fractions between 5% and 10% through which the compression properties are still comparable to those of unfilled systems.^[^ ^61f^ ^]^

**Figure 8:**
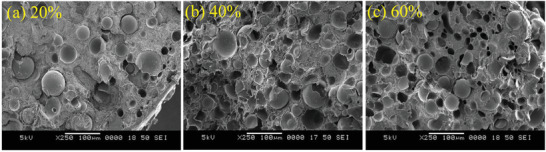
Cryofractured micrographs of HDPE filaments containing cenospheres of a) 20, b) 40, and (c) 60 vol%. Reproduced with permission.^[^ ^61e^ ^]^ Copyright 2019, Elsevier.

**Figure 9:**
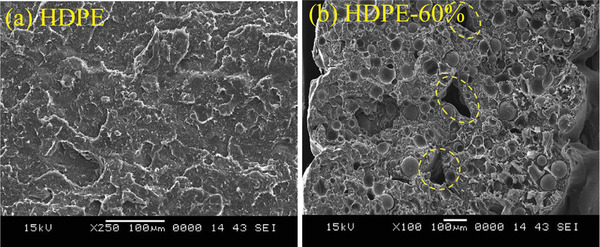
Cryofractured micrographs of 3D printed a) HDPE and b) HDPE with 60 vol% cenospheres. Air gaps are observed in printed parts with 60 vol % cenospheres. Reproduced with permission. ^[^ ^61e^ ^]^ Copyright 2017, Elsevier.

The compounds containing hollow micro‐balloons (fly ash cenospheres) were later on prepared with high density polyethylene (HDPE) and cenospheres contents of 20, 40 and 60 vol%.^[^ ^61^, ^62^ ^]^ **Figure** **8** shows the cryofractured micrographs of the filaments containing cenospheres of 20, 40 and 60 vol% in HDPE. The filaments were then prepared to produce their 3D printed structures. Using these hollow fillers, they showed that the complex viscosity and storage modulus were increased throughout the entire frequency range and more specifically at low frequencies due to the introduced solid‐like structure. Despite the use of such high content of cenospheres, the final 3D printed structures revealed void fractions only up to only 10%. It was observed that by using cenospheres and increasing content, the tensile modulus of the 3D printed structures increased, but the tensile strength, ductility, and toughness were dramatically decreased. This was confirmed to be due to the formation of large air gaps between the layers in the printed parts with cenospheres. **Figure** **9** compares the 3D printed HDPE and HDPE with 60 vol% cenospheres, clearly showing the printing quality and the existence of voids among layers.

**Figure 10:**
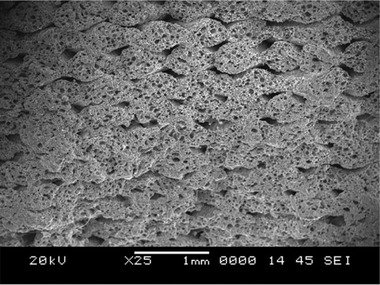
SEM cryofractured micrographs of 3D printed HDPE with 60 vol% GMBs showing the air gaps formed among the printed layers. Reproduced with permission. ^[^ ^61d^ ^]^ Copyright2020, American Chemical Society.

Quite similar behavior was also reported in another study of the same group using hollow glass micro‐balloons (GMBs) where void fractions up to 30% were obtained.^[^ ^61^, ^63^ ^]^ Similar to the previous study, the tensile and flexural mechanical properties of the printed structures were not improved by the addition of GMBs due to the formation of voids and air gaps between the printed layers (**Figure** **10**). This is because the final density of the printed cellular structures is still quite high despite the high contents of low‐density microspheres.

### 3.3 Post‐Foaming of Pre‐Formed Solid Structures

**Figure 11:**
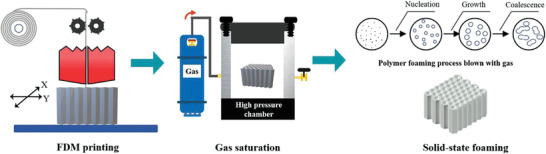
Schematic foam preparation of the printed structures through solid‐state foaming. Reproduced with permission. ^[^ ^64f^ ^]^ Copyright 2021, Elsevier.

Another simple route to combine additive manufacturing is post‐foaming 3D printed structures. The pre‐manufactured, not necessarily cellular structure, is first saturated with blowing agent and subsequently foamed. A few studies have used solid‐state CO_2_ batch foaming setups.^[^ ^64^ ^]^ The schematic of such a simple lab‐scale approach is illustrated in **Figure** **11**. Hu et al.^[^ ^64f^ ^]^ revealed that the microcellular foamed structure of TPU honeycombs improved the energy absorption efficiency up to 0.40, whereas that of the corresponding unfoamed honeycombs showed only 0.32–0.38 efficiency. Moreover, microcellular TPU honeycombs demonstrated an improved elasticity and better elastic recovery compared to their unfoamed counterparts.

Since the aforementioned lab‐scale foaming approach could not be extended to the industrial scale, concurrent foaming and printing should be developed to simultaneously reach foamed printed structure that could also be applied at industrial scale. Since this technology is not yet established, it is expected to be the future trend of 3D printing and foaming technologies.

### 3.4 In Situ Foaming of Filaments Containing Blowing Agent

**Figure 12:**
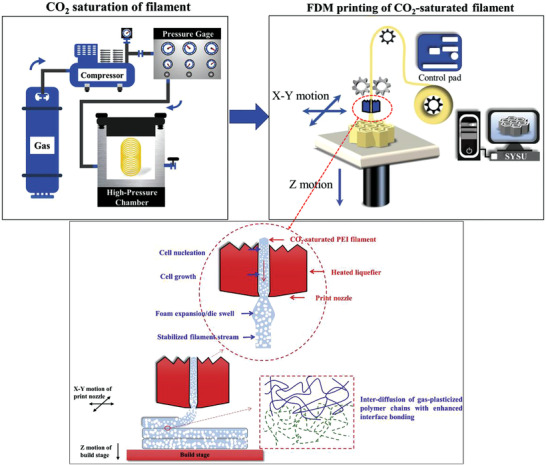
Schematic of foam 3D printing of the pre‐saturated filaments. Reproduced with permission.^[^ ^65d^ ^]^ Copyright 2020, Elsevier.

Very recently, few attempts introduced the development of filaments containing CBAs or saturated filaments with PBAs to induce foaming during the printing phase. Foaming occurs during printing with the pressure drop at elevated temperatures leading to thermodynamic instability.^[^ ^65^ ^]^ **Figure** **12** shows the schematic of such in situ foam 3D printing of pre‐saturated filaments with CO_2_ and concurrent printing and foaming. As seen, cell nucleation and growth occur at the nozzle, where sudden depressurization occurs under certain printing temperatures.

Although it is still in its early research stage and has not been properly established yet, this foam 3D printing approach seems to be the most practical route to successfully manufacture foamed printed structures. Therefore, it could be considered a serious future trend for both additive manufacturing and foaming technologies, which could be a promising alternative to foam injection molding.

In this approach, the first major concern is to obtain saturated filaments that could maintain the dissolved blowing agent until printing. Therefore, the use of PBAs such as N_2_ that has a very low solubility and high diffusivity in thermoplastics seems impossible. In contrast, the use of hydrocarbons with high solubility and very low diffusivity could be promising to achieve stable saturated filaments. These blowing agents are also used to produce saturated polystyrene micro pellets that are available under ambient conditions to manufacture expanded polystyrene bead foam.^[^ ^10a^ ^]^ However, as discussed in Section 2.1.3., the use of hydrocarbons is increasingly being avoided and replaced by environmentally friendly blowing agents such as N_2_ and CO_2_. Hence, in foam 3D printing attempts, CO_2_ has mainly been used to saturate filaments although it is still not as soluble in polymers as hydrocarbons. It is worth noting that the incorporation of CBAs could be a breakthrough in the production of high‐density 3D printed foams, as the difficulties of preserving dissolved gas in filaments may not be a major concern. However, it is also known that the use of CBAs is not as efficient as that of PBAs.

**Figure 13:**
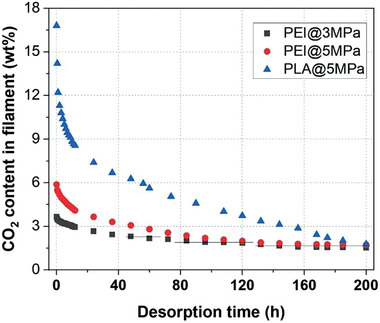
Desorption curves of the CO_2_‐saturated PEI and PLA filaments over time. Reproduced with permission.^[^ ^65d^ ^]^ Copyright 2020, Elsevier.

Overall, the polymer type and its molecular structure influence the dissolved blowing agent content in filaments and its preservation until printing.^[^ ^66^ ^]^ For instance, as shown in **Figure** **13**, while polyetherimide (PEI) with high *T*_g_ (≈217 °C) and low gas diffusivity dissolves lower CO_2_ content, the CO_2_ desorption in this polymer is slower over time compared to that in PLA, although PLA has a lower *T*_g_ of around 60 °C and dissolves larger CO_2_ contents. Such CO_2_ desorption behavior over time might become slower in PLA grades with higher molecular weight or branched structures or those with higher degree of crystallinity. In such structures, the gas diffusion could be retarded due to the higher molecular entanglements, which could encapsulate more CO_2_ contents after saturation.^[^ ^67^ ^]^

**Figure 14:**
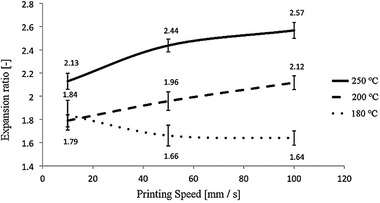
Effects of printing temperature and printing speed on the expansion ratio of the foamed printed structures. Reproduced with permission.^[^ ^65c^ ^]^ Copyright 2017, Wiley‐VCH.

**Figure 15:**
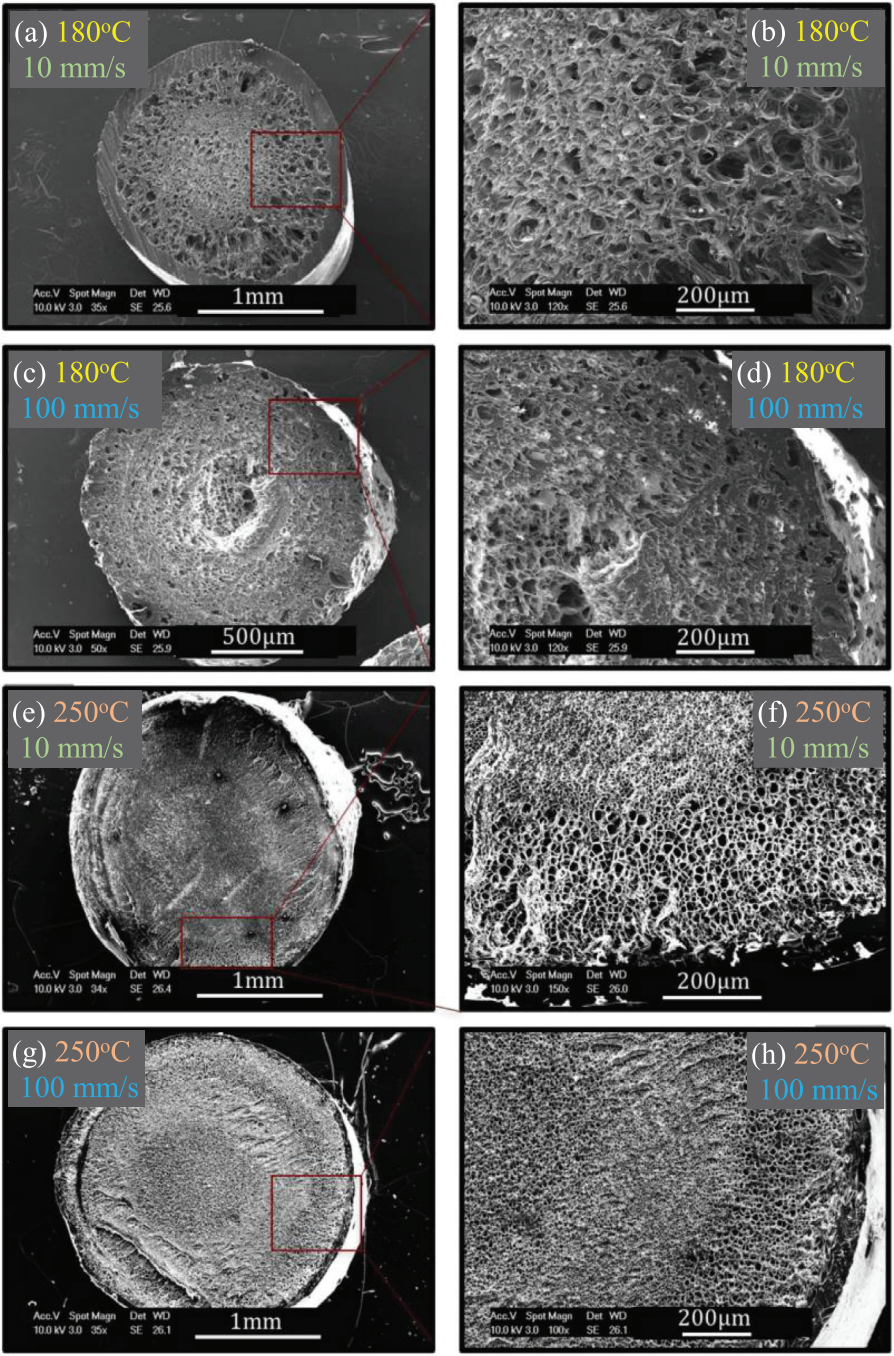
SEM images depicting the cellular structure of the filaments at extreme printing temperatures (a‐d: 180 °C and e‐h: 250 °C) and speeds (a,b,e,f: 10 mm s‐1 and c,d,g,h: 100 mm s^−1^). Reproduced with permission.^[^ ^65c^ ^]^ Copyright2017, Wiley‐VCH.

In one of the first studies, CO_2_‐saturated PLA filaments containing around 14 wt% CO_2_ were prepared and in situ foam 3D printing was conducted at different printing temperatures (i.e., 180, 200, and 250 °C) and speeds (i.e., 10, 50, and 100 mm s^−1^).^[^ ^65c^ ^]^ **Figure** **14** illustrates how these parameters can affect the expansion of the foamed printed structures. **Figure** **15** also depicts the obtained cellular structure at the cross sections of the filaments. It was shown that heat transfer at 180 °C was insufficient for cell growth and inducing expanded foamed structure. The foam expansion was, however, promoted more noticeably at 250 °C due to the facilitated cell growth subsequent to cell nucleation. At 200 and 250 °C, the increased printing speed also resulted in higher foam expansion due to the induced higher depressurization rate.

According to the SEM images shown in Figure 15, inhomogeneous cellular morphologies were obtained due to the insufficient heat transfer at 180 °C, while at low printing speeds (10 mm s^−1^) thicker solid skins were formed on the surface of the filaments. Therefore, the low cooling rate and poor heat transfer resulted in cell size increase from the center of the filament toward the skin. In contrast, at higher printing speed (100 mm s^−1^), the faster depressurization caused cell coalescence at the filament core and the formation of a hollow‐like structure.

When the printing was conducted at 250 °C, more homogeneous foam morphologies were observed especially at higher printing speeds. While at low printing speed (10 mm s^−1^) larger and less homogeneous cells are observed, a finer cell structure with homogeneous gradient from core to skin could be observed at high printing speed (100 mm s^−1^). This is due to the increased depressurization rate which promotes the cell nucleation and homogeneity of the foam structure. As seen, the solid skin layer is also quite thin in this sample due to the homogeneous foaming over the whole filament cross section. Similar effects of processing parameters, including the printing temperature and speed, on the foaming behavior of CO_2_‐saturated ABS printed structures were also confirmed by Dugad et al.^[^ ^68^ ^]^

**Figure 16:**
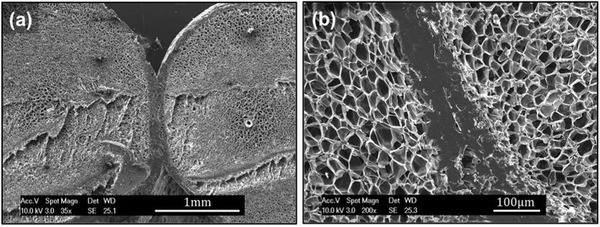
SEM images of layer‐by‐layer deposition of foamed filaments. a) Overview, b) magnified welding structure. Reproduced with permission.^[^ ^65c^ ^]^ Copyright 2017, Wiley‐VCH.

**Figure** **16** shows the welding (i.e., sintering) quality among the printed foamed layers when the printing was conducted at 250 °C and a speed of 100 mm s^−1^. As seen, the foamed PLA filaments could properly be welded between solid skin layers, where no cellular structure was induced.^[^ ^65c^ ^]^

**Figure 17:**
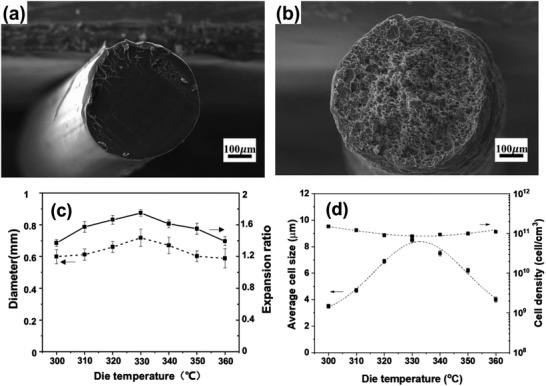
Cross‐sectional SEM images of a) solid and b) foamed PEI filaments as well as c) filament diameter and expansion ratio and d) average cell size and cell density of the foamed PEI as a function of printing nozzle temperature (i.e., die temperature). Printing speed was fixed at 60 mm s^−1^. Reproduced with permission.^[^ ^65d^ ^]^ Copyright 2020, Elsevier.

**Figure 18:**
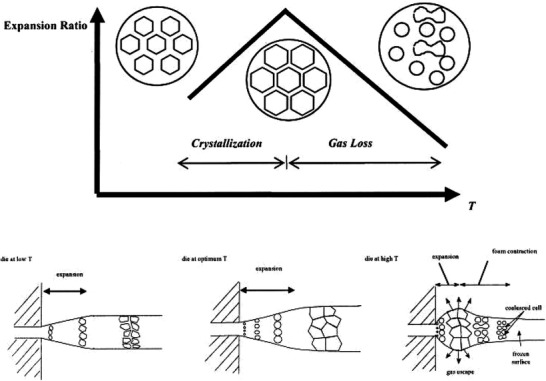
Effect of die temperature during extrusion foaming on the cell growth and foam expansion. Reproduced with permission.^[^ ^15c^ ^]^ Copyright 2004, Wiley‐VCH.

Later on, the group of Zhai^[^ ^65d^ ^]^ confirmed that microcellular structures could be formed by printing of CO_2_‐saturated PEI filaments. As illustrated in Figure 13, the CO_2_ within the saturated PEI showed an extremely slow diffusivity. Hence the stable CO_2_ content in PEI more effectively caused the formation of cellular foamed printed structures in which the cell sizes were below 30 µm.^[^ ^14c^ ^]^ **Figure** **17**a,b illustrates the cross section morphology of solid PEI and CO_2_‐saturated PEI filaments after foaming. The dependence of filament diameter and the expansion ratio as well as the cell size and the cell density on the printing nozzle temperature (i.e., die temperature) are also depicted in Figures 17c and 17d, respectively. The change of printing temperature from 300 to 360 °C reveals that there is also an optimum printing temperature at which foamed structure with higher expansion could be obtained. This is similar to extrusion foaming (**Figure** **18**) where below a certain die temperature, the high rigidity or crystallization of the extrudate foam could suppress the cell growth and the foam expansion, whereas beyond such optimum temperature, the low melt strength causes the cell coalescence.^[^ ^15c^ ^]^

**Figure 19:**
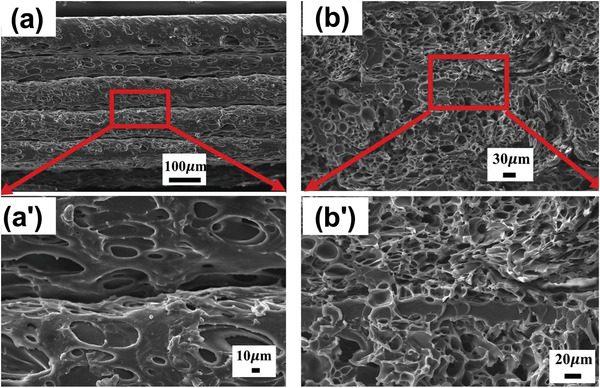
SEM micrographs of the stacked printed PEI foamed filaments a,a′) at the surface level and b,b′) at the cross section. Reproduced with permission.^[^ ^65d^ ^]^ Copyright 2020, Elsevier.

**Figure** **19** also shows the welding behavior of the stacked foamed PEI filaments after printing. As seen from the surface and cross‐sectional points of view, the foamed filament layers appear to be properly welded together, although mechanical testing should be performed to better examine. In a later study, the same group^[^ ^65b^ ^]^ showed that the printed PEI foamed structures revealed a high compression strength and modulus in the range of 24.7–54.7 and 187.5–438.8 MPa, respectively.

## 4 Challenges and Future Trends in Foam 3D Printing

As discussed in Section 3.4, in situ foam 3D printing has recently been focused and developed based on printing of saturated filaments. In this approach, the major concern is how to obtain blowing agent saturated filaments without having a loss of the dissolved blowing agent until the printing stage. CBAs could also be used within filaments although they are not efficient foaming agents as PBAs. In general, the polymer type and its molecular structure are important elements to determine the blowing agent dissolution degree and its preservation within the saturated filaments. This means that, based on current knowledge, not all thermoplastics are suitable for in situ foam 3D printing due to their gas solubility differences. Therefore, extensive studies should still be conducted on this new in situ foam 3D printing approach to overcome the noted limitations. Therefore, any breakthrough to develop concurrent foam printed parts will be a great success for the future of additive manufacturing and foaming technologies.

**Figure 20:**
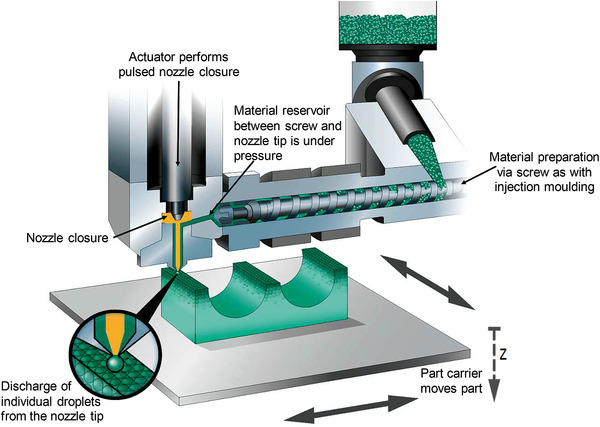
The schematic of freeformer 3D printing machine. Reproduced with permission. Copyright 2022, ARBURG.

Considering the aforementioned limitations of in situ foam 3D printing, another approach can also be proposed by the authors of this article. Inspired by extrusion foaming or foam injection molding technologies, it is believed that a filament free approach could be developed to print foam structures in a desired shape. In other words, a future perspective of concurrent foam 3D printing could be proposed by direct printing of a saturated polymer/gas mixture. Technologies like Freeforming^[^ ^29^ ^]^ or FGF could be used and modified to tackle foaming while printing, although studies have not been found on this proposed approach yet. Indeed, the commercially available freeformer machine (**Figure** **20**) that is designed to manufacture filament free 3D‐printed structures could be modified to foam in a manner similar to extrusion foaming and foam injection molding by injecting the blowing agent directly to the extruder barrel.^[^ ^1^, ^21^ ^]^ Therefore, through the generation of thermodynamic instability (i.e., depressurization) at the printing die nozzle, the foaming of the printed parts could simultaneously occur. This approach with the required machinery modifications could be a future alternative to develop filament free foamed 3D printed structures while retaining a high percentage of dissolved blowing agents right before the printing phase. In this approach, N_2_ with high diffusivity and high cell nucleation power could also be utilized to manufacture high‐density foams with desired 3D geometries similar to that in foam injection molding process. The modifications on this machinery could be considered as a new foam 3D printing alternative technology besides other common foaming technologies. Specifically, the capability of manufacturing 3D structures could enable the replacement of foam injection molding with this proposed foam 3D printing technology. Still in this approach, the welding among the foamed layers could be the major concern and extensive research requires to be conducted on identifying possibilities, difficulties, and breakthroughs.

## Conflict of Interest

The authors declare no conflict of interest.
